# Movement of avian predators points to biodiversity hotspots in agricultural landscape

**DOI:** 10.1098/rsos.231543

**Published:** 2024-01-10

**Authors:** Paweł Mirski, Jaan Grosberg, Thea Kull, Pelle Mellov, Grete Tõnisalu, Vivika Väli, Ülo Väli

**Affiliations:** ^1^ Institute of Agricultural and Environmental Sciences, Estonian University of Life Sciences, Kreutzwaldi 5D, Tartu 51006, Estonia; ^2^ Faculty of Biology, University of Białystok, Ciołkowskiego 1J, Białystok 15-245, Poland

**Keywords:** farmland, biodiversity monitoring, bioindicators, birds of prey, movement ecology

## Abstract

Global agricultural landscapes are witnessing a concerning decline in biodiversity, and this trend is predicted to persist. To safeguard these biodiversity-rich areas, it is crucial to pinpoint hotspots effectively. In doing so, we used various species of avian predators as suitable sentinel animals due to their mobility and dependence on prey diversity and abundance. Between 2019 and 2021, we tracked 62 individuals from four bird of prey species using GPS loggers in Estonian farmland. Dividing the study area into 50 m grids and overlaying them with tracked individuals' locations enabled us to differentiate between hotspots of their activity and control sites. We conducted surveys on amphibian, bird, small mammal and plant abundance and diversity to determine if avian predator activity hotspots correlated with overall biodiversity. Our findings revealed significantly higher diversity and abundance in the surveyed groups within activity hotspots compared to control sites. These hotspots continued to be frequently used by raptors in the subsequent year, albeit not two years later. In conclusion, multispecies GPS telemetry of avian predators emerges as an objective, dependable and spatially accurate biodiversity indicator. With the accumulation of movement data, we anticipate increased interest and adoption of this approach in biodiversity monitoring.

## 1. Introduction

In recent decades, agricultural landscapes have been subject to rapid transformations that have led to a degradation in their quality [1–3], consequently impacting biodiversity [4–6]. Furthermore, future projections indicate a continuous decline in the quality of agricultural ecosystems, particularly with regard to croplands and grasslands, due to intensification [4,7]. In order to safeguard farmland biodiversity and enhance our environmental policies concerning agriculture, it is imperative to pinpoint and quantify biodiversity within agricultural landscapes and investigate the factors that promote high species diversity.

Bioindicators stand as an efficient tool for effectively monitoring the distribution of farmland biodiversity. Various organisms have been used for this purpose, including birds [8], bats [9] and ants [10]. Nonetheless, distinct taxonomic groups exhibit varying responses to the primary threat of agricultural intensity, contingent upon the spatial scale of investigation [11]. Consequently, we are still in the process of formulating an appropriate bioindicator for sustainable farming [12]. It is challenging to identify universal bioindicators for such a dynamically evolving landscape, which can undergo rapid physiognomic alterations from one week to another (e.g. during ploughing, harvesting or mowing) as well as shifts in landscape composition from year to year (e.g. due to changes in crop distribution or crop rotation). Furthermore, patterns of farmland biodiversity are neither constant nor timeless but exhibit seasonally dynamic variations [13,14] and may hinge on microenvironments, ecotones, and even singular landscape elements [15,16].

Taking the aforementioned considerations into account, it becomes crucial to explore alternative and unconventional methods of bioindication to identify: (1) bioindicators that transcend geographical boundaries and are not tied to single species, (2) bioindicators that offer spatial precision to match the intricate mosaic patterns of farmland and reveal its critical components, and (3) bioindicators capable of providing nearly continuous temporal data, capturing the seasonal dynamics of agricultural cycles.

Possible candidates that meet the above criteria are avian predators, a group that has already been frequently considered reliable surrogate species [17]. The principal assumption underpinning their utility lies in their positioning at the upper level of the food chain, relying on other organisms as their primary source of energy. The latter is usually the small- and medium-sized prey spanning various taxa, including insects, amphibians, reptiles, mammals and birds [18]. Consequently, they represent potential indicators of areas supporting high diversity or abundance across various prey taxa. Studies conducted so far have tested this assumption by investigating the distribution of avian predators at the landscape scale [17]. The bulk of these investigations has yielded significant correlations between the distribution of raptors and the diversity of organisms, encompassing taxa unrelated to their prey base, such as trees, fungi, and butterflies [19–23]. Nevertheless, some studies have found no discernible advantage in utilizing raptors as biodiversity surrogates [24,25], while others have critiqued conclusions regarding the indicative value of raptors, attributing discrepancies to methodological biases inherent in the studies conducted to assess this [26,27]. It is noteworthy, however, that all above mentioned studies have generally operated at a relatively coarse scale, limiting their efficacy in identifying drivers of ecosystem quality that elucidate biodiversity dynamics in fine detail [17].

With the advent of advanced animal tracking devices, the field of movement ecology has witnessed rapid expansion in recent decades. Only more recently, it has been recognized as a valuable tool for investigating broader aspects of biodiversity [28,29]. Animal movements are tightly linked to spatial patterns of habitat structure and resource availability. Consequently, tracking wild animals facilitates the collection of a substantial volume of crucial spatial data concerning landscape utilization [30,31]. This approach offers far more comprehensive insights than single observations obtained through presence-only data, which have traditionally served as the foundation for most biodiversity surveys. Such data suffer from numerous biases, including biased site selection, variable detectability among species, habitat-dependent detectability, temporal patterns of habitat use by different species, observer presence effects and several others [32,33]. Furthermore, in conventional surveys, aligning animal observations with their specific habitats can prove challenging. For instance, many avian species may be easiest to detect in their display locations, which might differ from their critical foraging sites, thereby affecting species-specific detectability rates [34]. Conversely, GPS tracking furnishes spatial data with an accuracy down to even metres or tens of metres and temporal coverage encompassing entire annual and diurnal cycles. Crucially, tracking the movements of free-ranging wild animals offers an insider's view of their landscape utilization, including time spent in particular patches and the frequency of returns, enabling the quantification of spatial landscape quality [30]. Importantly, this approach minimizes observer influence [35] and is nearly devoid of detectability errors [34].

In summary, avian predators are considered effective bioindicators, and tracking them should result in fine-scale, extensive datasets that mirrors habitat structure and prey distribution. In this context, an additional advantage of using raptors as sentinels is their exceptional mobility. Medium-sized avian predators can cover daily distances that easily exceed the boundaries of their home ranges [36]. They are not constrained by geographical distances or obstructed by anthropogenic or natural barriers, which are often encountered by large mammals. Furthermore, their strong site fidelity, exhibited by both sedentary and migratory species, ensures that they are intimately acquainted with the distribution of resources within their limited areas [37]. This characteristic enhances their efficacy as indicators of ecological conditions and enables the collection of fine-scale GPS data over large areas (landscape scale). In the light of these advantages, we conducted a comprehensive multispecies GPS telemetry study on avian meso-predators to assess their movement patterns as a broader indicator of biodiversity hotspots. We assumed that the spatial utilization patterns of different raptor species mirror the diversity and abundance of various prey species, thus rendering multispecies telemetry a potential tool for identifying biodiversity hotspots. Furthermore, we propose that these hotspots of prey diversity and abundance are also characterized by a greater diversity of plant species, signifying a high-quality habitat conducive to overall biodiversity. Finally, since biodiversity hotspots are inherently linked to patches of superior ecosystem quality, we anticipate that many of these sites will exhibit slow rates of change, ensuring the persistence of their positive effects on biodiversity over the years. Consequently, avian predators are likely to revisit these hotspots in subsequent seasons.

## 2. Methods

### 2.1. Study area

**Figure 1.**
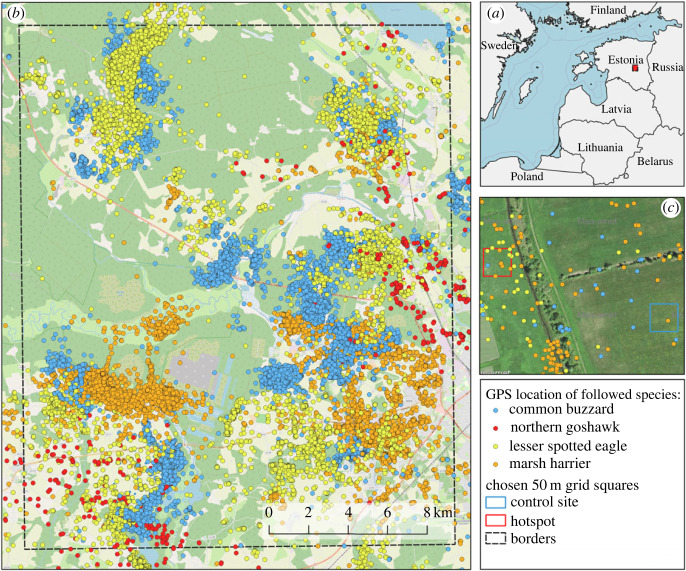
Location of the study (*a*), GPS telemetry dataset on 62 individuals of four bird-of-prey species (*b*), and an example (*c*) for designating hotspots versus control sites in agricultural landscape of southern Estonia.

This study was conducted in the central region of Estonia, situated in northeastern Europe, within a flat lowland area that borders the nemoral and boreal ecological zones (figure 1*a*,*b*). The study plot, spanning 20 km by 25 km, occupies a mosaic landscape characterized by a mixture of farmland and natural open habitats, including river valleys and peat bogs, interspersed with forested areas. The distribution of land cover in this plot exhibits a ratio of 45% forested land to 55% open land. Notably, a large forest complex, along with several medium and small forest complexes, predominantly occupies the northwestern portion of the plot.

The majority of the open landscape within this region is covered by farmland, encompassing approximately 46% of the total study area. Within this farmland, around 81% is used for arable crops, 16% serves as grasslands, and the remaining 3% is allocated to fruit orchards and various other types of agricultural cultures (Estonian Agricultural Register Map 2019). The remaining land cover within the study area comprises natural river valleys dominated by reeds (3%), peat bogs and peat mines (3.5%) and waterbodies (1%). Human settlements account for 1% of the land cover in the region.

### 2.2. Study species

Among the 28 raptor species that breed in Estonia, we selected four species that were deemed suitable for assessing the quality of agricultural ecosystems. These species are also common and large enough to carry GPS telemetry devices. The chosen species were the lesser spotted eagle (*Clanga pomarina*), common buzzard (*Buteo buteo*), western marsh harrier (*Circus aeruginosus*), and northern goshawk (*Accipiter gentilis*). The first three species are dietary generalists, known for hunting a wide array of small prey, often targeting the most abundant or available options. Their prey includes small mammals (primarily rodents), small passerine birds, their eggs and nestlings, amphibians, lizards, and even various invertebrates [38–40]. By contrast, the northern goshawk is a dietary specialist, predominantly preying on other bird species [41], rendering it an apt surrogate species for evaluating the diversity and abundance of avian fauna [21,42].

The density of the common buzzard has been demonstrated as a predictive factor for assessing the diversity of other farmland bird species [43]. Additionally, the movement patterns of the lesser spotted eagle have been shown to benefit from landscape diversity [44] and reveal areas of functional heterogeneity within agricultural landscapes [45]. These areas are characterized by a high diversity of various animal taxa, aligning with findings in the literature [46]. While the western marsh harrier has not yet been extensively studied as a species indicating broader biodiversity, its relative, the eastern marsh harrier (*Circus spilonotus*, formerly considered the same species), has been established as a reliable predictor of marshland bird diversity [23]. An added advantage of harriers in bioindication lies in their low territoriality, enabling them to traverse landscapes freely, even in proximity to the nesting sites of their conspecifics [47,48]. Furthermore, their hunting predominantly occurs during flight, leading them to move extensively throughout the day and cover substantial distances. This characteristic facilitates the collection of large quantities of spatial data over extensive areas, enhancing their utility in bioindication efforts.

### 2.3. GPS telemetry dataset

In this study, the key data to acquire were the movement patterns of wild raptors. Starting from April 2019, we trapped individuals of four different species of birds of prey in their known breeding territories. We used large mistnets with stuffed specimens of large avian top predators (white-tailed eagle and eagle owl) to provoke the attack of the focal species near their nest sites and to catch them in the net. We managed to trap and track 62 individuals of four different species (electronic supplementary material, table S1). As many as 59 individuals were caught during this project, but we also used additional data from three lesser spotted eagles that were caught earlier in the same area and were still transmitting data [45].

We used 15–30 g GPS loggers with solar panels (Ornitela, Lithuania), which were selected according to the body mass of each studied bird so as not to exceed the 3% threshold that is currently considered as acceptable by many bioethics committees. GPS loggers were attached to birds as backpacks with Teflon ribbon that were sewn at the sternum. Handling time reached 30–60 min per individual. We recorded no mortality in the following months and no individual deserted its brood after its trapping and tagging. GPS data were collected at 3- to 60-minute intervals depending on the battery level of each individual's logger. Marsh harriers and lesser spotted eagles flew extensively and charged their tags through sunlight, so they mostly were able to acquire data with 5- to 10-minute intervals. Common buzzards and northern goshawks spent around 90% of their time perching and mostly under canopies [49]. Therefore, their charging ability was much lower, and GPS fixes were usually acquired in 10- to 60-minute intervals, depending on the individual.

### 2.4. Designation of raptors' activity hotspots and control sites

To test whether the raptors’ space use would lead us to biodiversity hotspots, we first had to designate the hotspots of raptor activity—i.e. sites of highest use by different species and different individuals followed by GPS telemetry. For comparison, we also chose control sites. To designate both, we divided the study area into 50 m × 50 m grids, drawn in QGIS 3.22 and subtracted the forest, waterbodies and peatland layers. The chosen grid size is a few times higher than the GPS positioning accuracy and precise enough to register selected habitat patches with sufficient detail.

Biodiversity surveys were conducted at activity hotspots and control sites as GPS-tagging data accumulated through the years (see below). Data on the first half of birds marked with GPS tags in 2019 (*n* = 35) were used to designate pairs of hotspots and control sites for the biodiversity survey (bird survey) carried out in 2020. Chosen activity hotspots were then completed with the subsequent years based on movement data from early spring 2020 (April) and late spring 2020 (May). Activity hotspots for biodiversity surveys in 2021 were chosen using data from all of 2020, early spring of 2021 (April), and late spring of 2021 (May) (see electronic supplementary material, table S2, for the number in each set). We considered a grid cell as an activity hotspot when at least three different individuals of two different species were using it in a given season (figure 1*c*). If more such grid squares were located in the adjacent position, we chose the square with the highest number of species recorded inside. Squares with obvious and isolated perching posts (such as single trees or pylons) were discarded, as at a given spatial scale it may attract birds of prey more as suitable perching or roosting site than as foraging site. When choosing between squares with the same number of species, we chose one with a greater number of individuals (with the highest priority) or observations (with secondary priority). To avoid choosing sites that were too close to each other (bird prey survey involved detectability radius of at least 100 m from the grid centre), we discarded other activity hotspots or control site in grid cells closer than 450 m from the chosen activity hotspot.

Control sites were designated as farmland areas with similar characteristics to the hotspots and physically accessible to the individuals we tracked. We selected them as pairs with an activity hotspot, always located 500 m from their centres. The procedure began with the extraction of centroids of activity hotspots and the creation of 500 m buffers around them. We initiated the search for a control site starting from the first grid square intersected by the buffer at a 0° azimuth from an activity hotspot, moving clockwise. The control site was the first suitable cell found, with the following exclusions: (1) cells that had more than one observation of tracked raptors in a given spring (to avoid similarities with hotspots), (2) cells that were closer than 500 m to already chosen activity hotspots and other control sites in the same season, and (3) cells containing landcover other than farmland.

Hotspots and control sites were designated using part of the tracking data in a given season or even utilizing data from the previous season. To verify the disparities in raptor activity at these sites, once the breeding season data were completed, we tallied the number of all observations, the count of different individuals and species, and the number of different days these sites were used by the tracked birds during the season, within each grid square (all done in QGIS 3.22). To accomplish this, we did not employ the raw dataset. Instead, we resampled the data into 15-minute intervals using the *amt* package in R [50]. This approach allowed us to objectively assess the accuracy of activity hotspot selections, irrespective of variations in data collection among the best-charging individuals.

### 2.5. Biodiversity surveys

We measured the species richness and abundance of amphibians, birds, and small mammals, as well as the species richness of vascular plants. The sample sizes differed between the studied groups because of large differences of labour in inventories of various taxa from 15 min bird counts to 48 h small-mammal trappings. However, the ratio between activity hotspots and control sites was always drawn in equal proportions.

We conducted the inventory of vascular plants in July–September 2020–2021. In total, we studied 120 50 m × 50 m plots (68 in 2020, 52 in 2021; electronic supplementary material, figure S1). In each plot, we assessed the species composition and the coverage of each species in five 1 m^2^ squares and estimated species richness as a total species list of all five. We also calculated the Jaccard index as a diversity measure to estimate the species similarity between the squares [51]. The plant species lists of five 1 m^2^ squares were compared in pairs, and the average Jaccard index was calculated for one plot. The smaller the average value of the Jaccard index, the greater was the difference in the species composition between the squares, and the greater was the species diversity in the 50 m × 50 m plot.

We conducted bird inventories in 74 plots in the first year and in the same number in the next year following the methodology of point counts [52]. We counted birds twice in a season (in the first half of May and in the first half of June) and averaged the numbers. The observer stayed at the centre of a 50 m × 50 m plot for 15 min and recorded all birds within a 100 m radius (electronic supplementary material, figure S2). Hence, the actual studied area was larger than the 50 m × 50 m plot because we expected that the observer may scare birds away from the plot.

We studied small mammals in 92 plots (46 plots per study year). The main dataset was collected by trapping small mammals in August and September. In each 50 m × 50 m plot, we conducted trapping in five 1 m^2^ squares (electronic supplementary material, figure S2). In each 1 m^2^ square, we placed a set of traps consisting of two snap-traps, two box-traps (Sherman large; 23 × 9 × 8 cm^3^), and one trap-hole (a cone made of a bottomless plastic bottle with a depth of about 25 cm and an upper diameter of about 10 cm). Each trap was baited with a piece of black bread. We trapped small mammals for two consecutive nights in each plot. We set the traps up in the evening and checked them in the next morning. We counted amphibians in the course of other inventories (birds, mammals, vascular plants) in a total of 92 plots (46 annually). Observers walked five 50 m long transects, counting all amphibians observed within 10 m stripe of the transect line.

### 2.6. Statistical analyses

Because the number of plots for biodiversity surveys differed between studied organisms, we could not compare activity hotspots and control sites in a single model. Instead, in R software we used generalized linear models with a binomial distribution for each explanatory variable (abundance or species richness of each prey group, plant species richness or similarity) separately. We built eight separate models with single predictor to test how each of them discriminated between activity hotspots (value 1) and control sites (value 0). To judge if abundance or diversity had greater impact to attract birds of prey, we calculated (using *rcompanion* package [53]) and compared the models in terms of variance explained and goodness of fit, estimated with McFadden's *R*^2^ and pseudo *R*^2^, respectively.

In order to check whether the effect of a hotspot in agricultural landscape lasts longer than one season, we checked if those sites were still attracting raptors, more than control sites in next years. The number of observations, individuals, species, and returning visits in following days per hotspot squares used by followed raptors were measured on the same plots between 2019 and 2022. Wilcoxon test was applied to compare each of above variables between hotspots and control sites, one and two years after they were chosen.

## 3. Results

**Figure 2.**
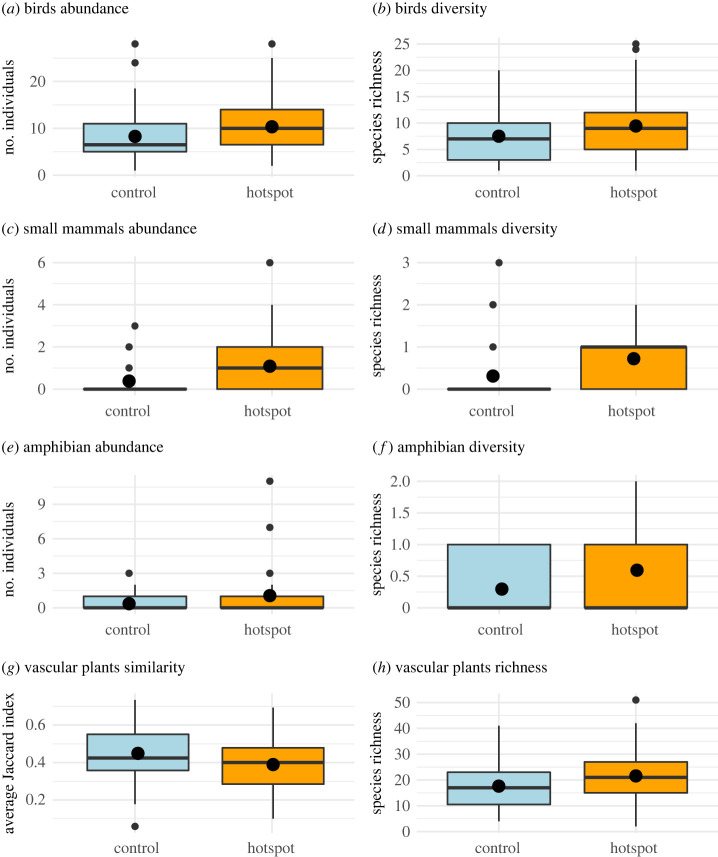
Comparison of abundance and species richness of birds (*a,b*), small mammals (*c,d*), and amphibians (*e,f*), as well as plant species similarity (*g*) and plant species richness (*h*) in Estonian farmland between hotspots of spatial activity of four different bird-of-prey species followed with GPS telemetry and control sites. Boxes represent the median (bold horizontal line), first and third quartile (hinges), minimum/maximum values (whiskers), mean (bold dot), and outliers (smaller dots).

Hotspots of raptor activity differed significantly from control sites, in terms of prey diversity and abundance (figure 2, table 1). Amphibians, birds, and small mammals exhibited greater species richness and higher abundance in activity hotspots compared to control sites. Moreover, plant species richness and similarity (expressed as the Jaccard index) differed between raptors' activity hotspots and control sites, as expected.

**Table 1.** Generalized linear models with binomial distribution comparing birds-of-prey space-use hotspots and control sites in terms of small prey species richness, abundance, and plant diversity in Estonian farmland. Significant differences are in bold.

predictors	*β*	CI	*p*	*n*	*R*^2^/pseudo *R*^2^	AIC
(intercept)	−0.01	−0.34–0.32	0.957		0.027	
bird abundance	0.40	0.07–0.77	**0.023**	147	0.36	202.25
(intercept)	−0.01	−0.34–0.32	0.949		0.025	
bird richness	0.37	0.04–0.73	**0.034**	147	0.35	205.0
(intercept)	0.10	−0.34–0.55	0.663		0.078	
small mammal abundance	0.79	0.27–1.41	**0.006**	92	0.62	121.59
(intercept)	0.07	−0.36–0.51	0.743		0.065	
small mammal richness	0.66	0.20–1.18	**0.008**	92	0.62	123.2
(intercept)	0.12	−0.31–0.58	0.585		0.055	
amphibian abundance	0.90	0.19–1.83	**0.035**	92	0.61	124.39
(intercept)	0.06	−0.37–0.49	0.786		0.049	
amphibian richness	0.56	0.12–1.04	**0.017**	92	0.61	125.18
(intercept)	0.05	−0.32–0.43	0.775		0.031	
plant similarity	−0.43	−0.83 – −0.05	**0.029**	117	0.49	161.08
(intercept)	0.04	−0.33–0.40	0.841		0.030	
plant richness	0.42	0.05–0.82	**0.030**	120	0.48	165.33

The generalized linear models explaining difference between activity hotspots and control sites in respect of small mammal richness and abundance explained 7–8% of the variance with each of these factors (table 1). The remaining models explained 3–6% of the variance, each with a single predictor for other fauna richness or abundance or with plant species richness and similarity. Prey abundance showed similar predictive value as prey richness. Most of the models showed high goodness of fit (measured with pseudo *R*^2^), except for models explaining bird abundance and diversity, which showed only moderate fit.

To study the durability of hotspots of raptor activity, we compared the hotspots and control plots identified in 2019 and 2020 with respect to the number of visits, individuals, and species of tracked individuals in the same sites in following years. Hotspots of raptor activity identified in 2019 were used by significantly more individuals and species, compared with control plots, also in the next year (2020), but not two years after (2021; table 2). Similarly, activity hotspots identified in 2020 were used significantly more often by a more diverse set of raptors than control plots in the following year (2021), but not the next year.

**Table 2.** Persistence of birds-of-prey space-use hotspots over time measured with variables describing space use and compared with Wilcoxon test between hotspots and control sites. Hotspots were designated with the use of movement data over a single season and compared with concurrent control plots one and two years after.

hotspot prediction	birds-of-prey hotspot predictors							
	no. of observations		no. of individuals		no. of species		no. of revisits	
	W	*p*	W	p	W	*p*	W	*p*
2019 to 2020	466.5	*	465	*	466	*	466.5	*
2020 to 2021	902	**	905	**	905	**	902	**
2019 to 2021	500	ns	499	ns	497	ns	497.5	ns
2020 to 2022	331.5	ns	336	ns	336	ns	333	ns

## 4. Discussion

Our movement-based approach provided a distinct advantage in pinpointing biodiversity hotspots with exceptional spatial precision. By contrast, prior studies assessing the bioindicative value of raptors primarily evaluated coarse landscape units ranging from 1 to 50 km^2^ [21,25,43,54]. In our study, we operated at a much finer scale, employing basic units of 0.25 ha for biodiversity surveys (and slightly larger areas for bird counts). This finer granularity likely contributed to our ability to detect higher levels of diversity in activity hotspots across all studied groups of organisms. Interestingly, at the fine scale of our study, bird diversity and abundance contributed less than small mammals and amphibians to raptors’ activity hotspots. This is most possibly also an effect of scale at which those prey animals operate. Birds, on average, are more mobile and require more space than the other mentioned taxa. Therefore, as a group, they are more affected by general landscape heterogeneity and are less sensitive to processes at patch level [55]. By tracking raptor movements with GPS accuracy, we were able to precisely identify locations where various avian predators detected sites rich in prey. This encompassed habitat selection at a remarkably detailed level, delving even deeper than the patch level and possibly included microhabitat diversity, landscape composition, and configuration patterns [45], factors often overlooked in coarse-scale analyses. This likely accounts for the disparities observed in other studies, carried out in coarse scale and therefore failed to establish a relationship between raptor presence and the diversity of taxa other than birds [21,23]. It is important to underscore that one key factor contributing to the success of biodiversity hotspot identification in our study was the multispecies approach to bioindication. A meta-analysis by Natsukawa & Sergio [17] demonstrated that the performance of apex predators as biodiversity indicators was enhanced when there existed at least a moderate ecological link between the predator and other taxa in the ecosystem. By employing multiple species, we multiplied the number of evaluated links between predators, prey, and their respective habitats, thereby bolstering the overall bioindicative capacity of raptors.

While there may be concerns that a generalist predator could be overly focused on one type of the most abundant prey or group of prey, our findings did not support this expectation. Prey diversity and abundance both explained observed differences with similar strength. Generalist predators exhibit adaptability in their prey selection, adjusting their choices according to the environmental conditions in their vicinity. For instance, widely distributed predators like the common buzzard and northern goshawk demonstrate the highest diet variability and the lowest dominance of single prey groups in heterogeneous environments [56] compared to managed landscapes [57,58]. Similarly, at a finer scale, raptors can explore foraging sites characterized by both high diversity and high prey numbers. Of course, even among generalists, some individuals may exhibit specialization in particular types of prey [59–61], and certain prey species may be more preferred due to higher rewards. In our study area, small mammals constituted a significant portion of the diet for the common buzzard [62], lesser spotted eagle [63–65] and western marsh harrier (Ü.V., unpublished data). This aspect did influence our results to some extent since the richness and abundance of small mammals emerged as significant predictors of hotspots in our study. Nevertheless, specialization in one type of prey can have adverse effects on raptor fitness when that particular prey becomes scarce [66]. This scenario is common in northern Europe, where many small mammal populations exhibit cyclic dynamics spanning three to five years [67,68].

The relationship between avian predators and their environment extends beyond mere predator–prey interactions. Our study revealed that raptors frequently utilized sites with higher plant diversity, characterized by greater total species richness and lower similarity of flora. This suggests that avian predators exploit areas with enhanced microhabitat diversity, which is often reflected in increased plant diversity. This selection aligns with findings in studies by Sergio *et al*. [20], Burgas *et al*. [22], Väli *et al*. [45] and Natsukawa *et al*. [42], reinforcing the significance of prey diversity and the capacity of raptors to identify diversity hotspots rather than simply focusing on sites abundant in prey. The variability in plant species fosters a greater number of links in the food web, creating opportunities for herbivorous, frugivorous, and granivorous species and ultimately benefiting the diversity of small prey.

Furthermore, our research demonstrated that these hotspots of raptor activity generally persist beyond the scope of a single season. Even after one year, hotspots remained more frequently visited by raptors than control sites, evidenced by a greater number of individuals, species and the total number of visits and revisits (different days of using). However, after two years, the differences became statistically insignificant. Undoubtedly, the conditions within agriculturally managed landscapes fluctuate from year to year, and the attractiveness of these hotspots may wane over time. Therefore, the use of movement data in bioindication remains reliable for at least one additional year, and possibly longer, until significant changes in land management practices occur. In more stable environments (such as forests, wetlands, mountains, etc.) we can expect more prolonged effects of hotspots, making multispecies movement approach to bioindication even more profitable.

Utilizing movement data in bioindication offers numerous advantages, as previously outlined. However, it is important to acknowledge that the availability, quality and quantity of movement data can be limiting factors. Acquiring movement data can be a costly endeavour, requiring expertise, specialized equipment and often special permits. The use of GPS tags may also have an adverse impact on studied individuals [69]. Therefore, employing movement data to identify biodiversity hotspots may entail more effort and limitations than conducting biodiversity surveys with lower sampling frequencies. The cost-effectiveness of this approach would likely depend on the sampling frequency, with movement data becoming advantageous when high-frequency data collection is necessary, such as on a weekly basis. Nevertheless, more comparative studies evaluating the cost- and labour-effectiveness of these approaches are needed.

Nonetheless, it is worth noting that in traditional biodiversity surveys, obtaining data with the same precision and objectivity as movement data is practically impossible. Hopefully, the number of scientific and conservation initiatives will continue to grow, with data-sharing becoming more widespread. Currently, the primary platform facilitating the sharing of such data is the Movebank repository, which houses over 6 billion animal locations and receives 3 million new data records daily [70]. As a result, the availability and quantity of movement data are expected to expand, creating more opportunities to use movement data in bioindication. This holds the promise of advancing our understanding and conservation efforts in the realm of biodiversity assessment.

## 5. Conclusion

The utilization of multispecies movement data from avian predators emerges as a reliable and valuable source of information for identifying biodiversity hotspots. Tracking the movements of generalist raptors afforded us an unparalleled insight into the most favourable farmland habitats for encountering a diverse array of small prey and flora. The chief advantage of incorporating movement data into bioindication lies in the objectivity inherent to such data and the capacity for high spatial precision, enabling the identification of critical sites for biodiversity. A notable trend in recent years is the proliferation of wildlife tracking studies, a development that augments the potential for utilizing movement data in bioindication and for investigating a wide range of ecological issues. The growing wealth of data generated from tracking the movements of various animal species offers a promising opportunity to enhance our understanding of biodiversity dynamics, ecological interactions, and conservation efforts. As technology advances and more researchers contribute to this field, the scope and impact of movement data in ecological research are poised to expand significantly, providing a powerful tool for addressing pressing environmental challenges and fostering a deeper appreciation for the intricacies of our natural world.

## Data Availability

The data of biodiversity inventories and tracking data are available via the Dryad Digital Repository: https://doi.org/10.5061/dryad.d2547d87n [71]. Supplementary material is available online [72].
